# A Large-Scale Seroprevalence of Epstein-Barr Virus in Taiwan

**DOI:** 10.1371/journal.pone.0115836

**Published:** 2015-01-23

**Authors:** Chao-Yu Chen, Kuan-Ying A. Huang, Jen-Hsiang Shen, Kuo-Chien Tsao, Yhu-Chering Huang

**Affiliations:** 1 College of Medicine, Chang Gung University, Taoyuan, Taiwan; 2 Division of Pediatric Infectious Disease, Department of Pediatrics, Chang Gung Memorial Hospital, Taoyuan, Taiwan; 3 Department of Laboratory Medicine, Chang Gung Memorial Hospital, Taoyuan, Taiwan; University of Hong Kong, HONG KONG

## Abstract

**Background:**

Epstein-Barr virus (EBV) causes a variety of clinical manifestations from asymptomatic infection to acute infectious mononucleosis in human. Moreover, the EBV infection is associated with malignancies. The large-scale EBV seroepidemiology across all age groups has been lacking in Taiwan.

**Methods:**

A total of 1411 serum samples were tested to examine the seroprevalence of EBV in 2007. The samples were collected during an island-wide seroepidemiological survey of vaccine preventable diseases in Taiwan. The enzyme-linked immunosorbent assay was performed to detect anti-EBV viral capsid IgG in sera. Demographic and personal health data were obtained by questionnaires.

**Results:**

The overall weighted seropositive rate of EBV was 88.5% (95% CI, 86.7%–90.1%). The seropositive rate of EBV reached 52.8% (95% CI, 44.0%–61.6%) in children aged 2 years, rapidly rose to 88.7% (95% CI, 79.0%–95.1%) in those aged 5–7 years and 93.0% (95%CI, 83.0%-98.1%) for those aged 14–16 years. Age and higher educational level were associated with the increased EBV seropositive rate.

**Conclusion:**

In Taiwan, people had the EBV infection early in life. Children under 7 years should be the primary target popution of public health measures in the future.

## Introduction

Epstein-Barr virus (EBV) belongs to the family of herpesviridae and serves as the dominant cause of acute infectious mononucleosis in young children and adolescents [1, 2]. Primary acute EBV infections are largely asymptomatic and the latent infection could persist for the lifetime [1]. In rare circumstances, chronic active EBV infections might lead to severe morbidities and death in previously healthy individuals [3, 4]. Some EBV infections have been associated with hemophagocytic syndrome, a severe inflammatory illness, characterized by prolonged fever, cytopenia, and liver dysfunction [5]. Moreover, the virus is causally linked to several malignancies, including Burkitt’s lymphoma, Hodgkin lymphoma, tumors in HIV-infected patients, and nasopharyngeal carcinomas [6]. Previous large-scale seroepidemiological surveys in Europe and United States showed that over 50% of adolescents would have been seropositive to EBV [7–10]. High EBV seropositive rate ranging from 60% to 93% was reported in some regional studies [11–17]. However, there has been a paucity of large-scale seroprevalence data of EBV from all age groups in Taiwan.

Several risk factors, including age [18], gender [18, 19], ethnicity [7, 18], country or region of residence [19], household educational level [18, 20], kissing [2], and smoking habit [20], have been associated with the EBV seropositive rate. The analysis of risk factors for the acquisition of primary EBV infection would be helpful in identifying those susceptible populations.

Vaccination along with other public health measures greatly reduces the burden of several infectious diseases in the modern era. Although an approved EBV vaccine remains unavailable, the protective effect of the experimental EBV vaccine against virus-induced lymphoma is shown in primate model. In human clinical trial, the EBV gp350 vaccine is efficacious against infectious mononucleosis in young adults [21, 22]. These indicate the potential of prophylactic EBV vaccine as the preventive tool against clinical illnesses and associated malignancies in the future. The seroepidemiological data would provide valuable information for primary clinicians and public health specialists about the ideal target population of and the clinical benefit expected from the candidate vaccine. Here, we conducted a large-scale antibody prevalence study to examine the age-specific EBV seropositive rate and identify the associated demographic, socioeconomic, and lifestyle risk factors in Taiwan in 2007.

## Materials and Methods

### Study samples and Ethic statement

In the study, 1411 human sera were selected from samples collected for one large public health survey in 2007. This survey was conducted to investigate the seroprevalence of vaccine preventable diseases in Taiwan and a total of 3552 blood samples were obtained between August and October of 2007, as previously described [23, 24]. Briefly, the survey was conducted in 16 randomly selected townships in four geographic regions (northern, suburban area of northern, central, and southern Taiwan) representing 95.1% of the Taiwanese population in 2007. The age-stratified sampling design was used and individuals visiting the official health centers for routine health checkup and/or immunization were invited to participate in the serosurvey [23].

To examine the EBV seroprevalence in the Taiwanese population, we selected residual samples from all age groups. Since the EBV seropositive rate was high in the children under the age of five, reaching 86% at 4-year-old age group, in the previous study in Taiwan [11], the majority of serum samples in the 0- to 4-year-old age group were included in the present study. For other age groups, we randomly selected the samples of residual sera. We therefore included 39 in 39 samples (100.0%) in the less than 1-year-old group, 90 in 95 (94.7%) in the 1-year-old group, 127 in 127 (100.0%) in the 2-year-old group, 92 in 117 (78.6%) in the 3-year-old group, 84 in 103 (81.6%) in the 4-year-old group, 71 in 310 (22.9%) in the 5–7-year-old group, 57 in 316 (18.0%) in the 8–10-year-old group, 55 in 276 (19.9%) in the 11–13-year-old group, 57 in 230 (24.8%) in the 14–16-year-old group, 78 in 322 (24.2%) in the 17–19-year-old group, 123 in 438 (28.1%) in the 20–29-year-old group, 129 in 258 (50.0%) in the 30–39-year-old group, 100 in 260 (38.5%) in the 40–49-year-old group, 91 in 260 (35.0%) in the 50–59-year-old group, 122 in 257 (47.5%) in the 60–69-year-old group, and 96 in 144 (66.7%) in the over 70-year-old group [23]. Briefly, 89.9% for those aged less than 5, 20.4% for those aged 5–10, 22.1% for those aged 11–16, 38.1% for those aged over 17 were selected in the study. The study was approved by the Institute Review Board of Chang Gung Memorial Hospital and all subjects provided written informed consent. Demographic and the relevant medical information were obtained by questionnaires. The serum samples were stored at -80°C.

### Laboratory methods

The commercial enzyme-linked immunosorbent assay (ELISA) kit (Euroimmun, Germany) was used to detect anti-EBV viral capsid antigen (VCA) IgG in sera. The sensitivity and specificity of the kit were evaluated using clinical serum samples from twenty adult volunteers and the EBV infection was pre-characterized with clinical manifestations, viral isolations, and anti-EBV VCA-IgG/VCA-IgM/EA immunofluorescence tests (Meridian Bioscience). With a cut-off value of 22 relative units (RU)/ml, the sensitivity and specificity of the ELISA kit in determining the EBV infection was each 100%. In addition, the reproducibility of the test was investigated by determining the intra- and inter-assay coefficients of variation (CV) using five clinical sera. The intra-assay CVs were based on five measurements for each serum on the same day and were between 2.86% and 6.81%. The inter-assay CVs were based on five measurements for each serum performed on five different days and were between 11.07% and 18.73%. The ELISA kit provided both acceptable intra- and inter-assay variability.

We performed the ELISA kit in accordance with the respective manufacturer’s instruction. Briefly, the plate was incubated with 100μl of diluted serum at room temperature for 30 minutes. After washing for three times, peroxidase-conjugated anti-human IgG was added and incubated at room temperature for 30 minutes. After another washing for three times, the development substrate was added and incubated at room temperature for 15 minutes. The reaction was stopped and the optical density was measured at 450nm. The kit provided ready-to-use positive controls (IgG, human), negative controls (IgG, human), and calibrators (IgG, human). Positive, negative controls, and calibration standards were assayed on each plate. The standard curve was established by calibrators and used to calculate virus-specific antibody concentration. The seropositivity was defined as the virus-specific antibody serum concentration of ≥22 RU/ml.

### Statistics

The data were analyzed with the SPSS Statistics, version 17.0 (2008 SPSS Inc, Chicago, IL, USA). The overall seropositve rate and the seropositive rate by gender were calculated using sampling weights to adjust for the sampling strategy in the study. In weighting, the distribution of the 2007 serosurvey population by gender and age was used as the reference [23]. Spearman rank correlation coefficient was used to examine the strength of correlation between the age and the seropositive rate. The difference of the seropositive rate between groups was analyzed by the χ^2^ test and Fisher’s exact test. Univariate logistic regression was initially performed to explore potential risk factors (age, gender, rural/urban region, educational level, smoking history, drinking habit, marriage history, or chronic medical conditions) associated with the seropositive rate. Significant variables further underwent age- and multivariate-adjusted logistic regression to establish the independent role of risk factors on the EBV seropositive rate. The strength of association was estimated by calculating the odds ratio with the 95% confidence interval. P value < 0.05 was considered to be significant.

According to previous studies, the children under the age of five were the major group of primary EBV infection. Therefore, we set the age group as per group each year for those under 5 years old, per group every 3 years for those between 5 and 20 year, and per group every 10 years for those over 20 years old. In the study, the official defintion published by the Directorate General of Budget, Accounting and Statistics, Executive Yuan, Taiwan government was used to distinguish between urban and rural regions in Taiwan in 2007. A region with both the population size of over 20,000 people and the population density of over 400 people per square kilometer was defined as the urban region. Others were taken as rural regions. The information about the population size and population density of the township in 2007 was based on the official database (available at http://www.ris.gov.tw/zh_TW/346). The drinking habit was divided into three grades: less than twelve times in lifetime, less than twelve times in one year, and more than twelve times in one year. The educational level included five categories: never been to the elementary school, a graduate of elementary school, a graduate of junior high school, a graduate of high school, and a graduate of university/college. The marriage (ever-married v.s. never-married) and smoking history (non-smoker v.s. smoker) were also analyzed.

## Results

The 1411 serum samples were obtained from 830 females and 581 males. Among them, 199 subjects (14%) lived in the rural area and 878 (62%) and 476 (34%) were from northern and southern Taiwan.

The overall weighted seropositive rate of EBV was 88.5% (95% CI, 86.7%–90.1%) in Taiwan, 2007. Weighted seropositive rates among females and males were 87.1% (95% CI, 84.6%–89.3%) and 75.6% (95% CI, 71.9%–79.0%), respectively.

The seropositive rate significantly increased with the age (*P* < 0.0001) (Table 1). Children under 1 year old had similar seropositive rate as that of children between the ages of 1 and 2 (*P* = 0.5231). However, the age-specific seropositive rate quickly rose in the early life. Among the 2-year-old children, 52.8% (95% CI, 44.0%–61.6%) of them had positive anti-EBV VCA IgG response. The EBV seropositivite rate further increased to 88.7% (95% CI, 79.0%-95.1%) in the age group of 5–7 years. For people aged 14–16 years, the seropositive rate of EBV reached 93.0% (95% CI, 83.0%–98.1%). During the adulthood, the EBV seropositive rate slowly increased to over 95% until the old age.

**Table 1:** Age-specific seroprevalence of EBV in Taiwan.

**Age (years)**	**Sample size**	**EBV seropositive rate (95% CI)**
<1	39	23.1% (9.2, 36.9)
1	90	30.0% (20.4, 39.7)
2	127	52.8% (44.0, 61.6)
3	92	69.6% (60.0, 79.2)
4	84	69.1% (59.0, 79.1)
5–7	71	88.7% (79.0, 95.1)
8–10	57	80.7% (68.1, 90.0)
11–13	55	89.1% (77.8, 95.9)
14–16	57	93.0% (83.0, 98.1)
17–19	78	96.2% (89.2, 99.2)
20–29	123	98.4% (96.1, 100.0)
30–39	129	99.2% (97.7, 100.0)
40–49	100	98.0% (95.2, 100.0)
50–59	91	95.6% (91.3, 99.9)
60–69	122	98.4% (96.1, 100.0)
>70	96	100% (100.0, 100.0)
Total	1411	82.3% (80.3, 84.3)

Abbreviations: EBV, Epstein-Barr virus; CI, confidence intervals.

The association between the risk factor and the seropositive rate was shown in Table 2. Age was clearly a significant risk factor for the antibody prevalence of EBV. The higher educational level also accounted for the EBV seropositive rate. Although gender was not a significant risk factor for the EBV seropositive rate, among seropositive subjects, females had a relatively higher mean anti-EBV VCA antibody level than males (135.8±53.5 v.s. 130.1±55.2 RU/ml, Student’s t test *P* = 0.0348). The residents in rural regions had a mildly higher EBV seropostive rate than those in urban regions (86.9%, 95% CI, 81.5%-91.3% v.s. 81.5%, 95% CI, 79.2%-83.7%), but the adjusted odds ratio for the EBV seropositive rate was not statistically significant. Neither the smoking history, the drinking habit, the marriage history, nor chronic medical conditions such as diabetes mellitus and hepatitis were associated with the EBV seropositive rate. The detailed data regarding demographics, potential risk factors for EBV infection and serologic results of the study subjects are shown in S1 Table.

**Table 2:** Risk factors for the EBV seropositive rate in Taiwan.

	**Age-adjusted OR (95% CI)**	**P value**	Multivariate-adjusted OR* (95% CI)	**P value**
**Age**			1.09 (1,05, 1.13)	<0.001
**Female/Male**	1.15 (0.84, 1.58)	0.369	1.23 (0.87, 1.75)	0.233
**Rural/Urban**	1.20 (0.73, 1.98)	0.481	1.32 (0.78, 2.24)	0.307
**Ever-married/Never-married**	4.56 (1.69, 12.30)	0.003	3.11 (0.95, 10.07)	0.060
**Non-smoker/Smoker**	0.21 (0,03, 1.60)	0.132	0.16 (0.02, 1.53)	0.113
**Drinking habit**				
Less than 12 times in lifetime	1 (referent)		1 (referent)	
Less than 12 times per year	1.71 (0.63, 4.66)	0.292	1.79 (0.57, 5.60)	0.321
More than 12 times per year	0.70 (0.15, 3.29)	0.649	0.42 (0.08, 2.31)	0.319
**Educational level**				
Uneducated	1 (referent)		1 (referent)	
Elementary school	2.62 (1.52, 4.51)	0.001	2.11 (1.13, 3.94)	0.019
Junior high school	4.21 (1.69, 10.50)	0.002	3.31 (1.18, 9.30)	0.023
High school	5.09 (2.05, 12.68)	<0.001	3.62 (1.29, 10.15)	0.015
College/University	7.80 (2.93, 20.80)	<0.001	5.26 (1.81, 15.26)	0.002

* The significant variables in univariate analysis, including age, gender, rural/urban region, educational level, smoking history, drinking habit, and marriage history, were all included in the multivariate regression analysis.

Abbreviations: EBV, Epstein-Barr virus; OR, odds ratio; CI, confidence intervals.

## Discussion

This study provided the up-to-date seroepidemiological data of EBV across all age groups in Taiwan. Among the Taiwanese children under 15 years old, the age-specific EBV seropositive rate in 2007 was similar to that in an earlier study conducted by Tsai et al about 20 years ago [11]. Both studies found that the age-specific EBV seropositive rate rapidly increased in early childhood and exceeded 80% before adolescence. An EBV seropositive rate of nearly 80% was also found in Chinese children under 10 years old, described by Xiong et al [17]. In contrast, a lower seropositive rate with around 50% was noted in the same age group in western countries [7–10] (Table 3). Firstly, Asian dietary culture, that has developed the distingushed style of chopsticks and the frequent occasions when mothers prematicate food for young children who are still unskilled with chopsticks, might predispose the transmission of EBV through the exchange of saliva among household members and partially explain the seroprevalence of EBV in younger children in Taiwan. In addition, the findings that demonstrated spontaneous oral secretions of EBV by seropositive adults and successful replications of EBV in human oropharyngeal epithelial cells would further reinforce the role of saliva transmission in the epidemiology of EBV infections [25–27]. Secondly, this discrepancy may be related with the difference in the age distribution of EBV-associated infectious mononucleosis [3, 28–30]. In western countries, infectious mononucleosis mostly affects adolescents and young adults. In contrast, the majority of infectious mononucleosis cases occurred before 5 years of age in Taiwan [3, 30]. Moreover, young children with primary EBV infection could have viral oropharyngeal shedding for as long as weeks or months [31]. These pediatric patients would spread viruses in households and daycare facilities and this might contribute to the increased risk of EBV infections in toddlers and preschoolers. Thirdly, asymptomatic or subclinical EBV infection could as well result in the seropositive status in an individual [32]. It remains unclear about the level of subclinical EBV infections and its role in the transmission of virus in the Taiwanese population. To better understand the interaction among the age of EBV acquisition, the development of either symptomatic or subclinical illness, and the risk of EBV-associated malignancies, further longitudinal studies addressing the epidemiology in the general population and the immune correlate of protection against virus should be conducted.

**Table 3:** The comparison of age-specific EBV seropositive rates among countries.

	**Country**	**Year**	**Sample size**	**Age (years)**			
				**≤2**	**3~9**	**10~19**	**>19**
Europe	Sweden [8]	1967–1968	552	-	-	56%	-
	United Kingdom [9]	1991	308	-	-	64%	-
	Sweden [10]	1977–1978	84	-	-	64%	-
		1997–1998	275	-	-	62%	-
		1998–2000	2561	-	52%	-	-
Asia	Japan [12]	1996–1998	202	-	59%	87%	98%
	China [13]	1984	94	79%	93%	100%	-
	Thailand [14]	1998	425	35%	84%	85%	-
	Bangladesh [16]	1996	452	51%	88%	85%	88%
	Taiwan [11]	1984	1350	38%	87%	99%	-
		2007*	1411	40%	79%	93%	98%
America	United States [7]	2003–2004	2849	-	57%	73%	-
		2009–2010	2149	-	50%	65%	-

* the present study

Abbreviations: EBV, Epstein-Barr virus.

Age is one significant risk factor for the EBV seropositive rate and this is also shown in the previous study [18]. However, gender does not affect the EBV seropositive rate in the present and other studies [7, 10, 16, 33]. We did note that seropositive females had a higher mean anti-EBV VCA IgG level than seropositive males, although their infection status of EBV could not be confirmed in the study. The similar phenomenon was described by other EBV serological studies [16, 34–36]. This is generally in accordance with the notion that females mount more vigorous antibody and cellular responses to infection or vaccination than males [37]. Although underlined mechanisms involving high EBV antibody level in females remain unclear, the link between the high level of anti-nuclear antigen (NA)-2, rather than the anti-VCA IgG level, and the risk of multiple sclerosis in females was noted by Ascherio et al [38]. The seroepidemiological pattern of infectious diseases could be affected by the public health policy, hygeine behavior, and socioeconomical status. In the study, we noted that residents in the rural regions had a mildly higher EBV seropositive rate than urban residents, but the adjusted odds ratio did not reach statistical significance. Similar results have been shown in other studies, although the definition of urbanization could vary in different countries and studies [20, 39]. An estimate of 78% of Taiwanese population reside in urban areas and the high urbanization rate might result from the decline of rural industry and the continuous flow of people from rural to urban areas, and this stiuation might as well partly explicate the high percentage of urban residents in the study [40, 41]. We noted that higher education level was associated with the EBV seropositive rate in Taiwan. The low maternal and household educational level being significant risk factors were previously reported, but the information about the effect of an individual’s educational level on the EBV acquistion was limited [7, 18, 20]. A cohort study among university students showed that 46% (110 in 241) of seronegative subjects experienced EBV seroconversion during the time in college. Among them, only one quarter had clinical presentations of infectious mononucleosis and penetrative sexual intercouse was found to be a significant risk factor for the seroconversion, suggesting the transmission of virus through the sexual activity [42]. In another study, similar percentage (46%, 66 in 143) of seronegative university students had primary EBV infection in a 3-year follow-up period, in whom as high as 77% presented as infectious mononucleosis and deep kissing was a significant risk factor [2]. These findings showed that intimate activities might be associated with primary EBV infection in the college life. Nevertheless, futher prospective and follow-up study will be needed to determine whether other factors, such as the level of personal hygiene and the age of sexual debut, are responsible for the assoication between the elementary and high school life and the EBV seropositivity in Taiwan.

There were several limitations in the present study. Firstly, this was a cross-sectional study and the one-time sample was collected for each subject. Besides, only IgG anti-VCA response was examined in the study. Upon acute primary EBV infection, anti-VCA IgM antibodies are usually produced transiently in the early course of illness and disappear within 4 to 6 weeks [43, 44]. In contrast, anti-VCA IgG antibody response appears later, peaks around 2~4 weeks after illness onset, and remains detectable lifelong. Those in the early stage of primary EBV infections generating antibodies of only VCA-IgM type might therefore be overlooked in our serosurvey [17, 43–45]. Regarding anti-EBNA-1 IgG response, it is not seen in the acute phase of infection, but appears about 2~3 months after the illness onset and basically lasts through a person’s life [32, 44]. Combining the information about anti-EBV VCA IgG, anti-EBV VCA IgM and anti-EBNA-1 IgG antibody responses could be helpful to determine whether an individual has either primary acute or past infection, but this is not the main goal of current study [17, 43]. Since the measurement of anti-VCA IgG response generally has an equivalent value of detecting a past infection as anti-EBNA-1 IgG response and some individuals might either be anti-VCA IgM nonresponders or have lost anti-EBNA response years after acute primary infection, we choose to detect anti-VCA IgG response in sera in the study and this has also been applied in other EBV seroepidemiological studies [7, 16, 18, 20, 43]. Secondly, the EBV-specific antibody response was examined solely by the ELISA assay in the study. With regard to EBV-specific serological assays, indirect immunofluorescent assay (IFA) and western blots were two other established methods [7, 34, 43, 45]. Traditionally, IFA is the reference standard for the serodiagnosis of EBV infection [43, 46]. However, the IFA method is time-consuming and requires experienced technicians for fluorescence image reading and might not be suitable for the analysis of a large number of samples. Commercial enzyme immunoassays using native antigens demonstrated high concordance with the conventional IFA method in EBV serodiagnosis [47]. The ELISA reagent wells applied in the study were coated with a mixture of purified native EBV capsid antigens derived from EBV-infected B cell lysate (information available at http://www.euroimmun.ch/uploads/media/EI_2791_D_UK_A02.pdf) and there was 100% aggrement between the ELISA and IFA methods in clinical serum samples (refere to the method section). Besides, the enzyme immunoassays could be performed in the automated system, easy to handle, and produce a robust readout to interpret. Western blot analysis allows the detection of EBV-specific antibodies against all relevant viral antigens in one test, and the result is highly specific and valuable to confirm the EBV infection status [7, 43]. However, the western blot assay is laborious and less sensitive than the enzyme immunoassay. Therefore, the western blot assay is rarely utilized to screen for EBV infection in the large-scale serosurvey. Thirdly, in the study, higher percentage of samples from childen under 5 years old were included in the analysis than those of other age groups. The respective percentage of samples in the present study therefore might not reflect the actual size distribution of different age groups in the Taiwanese population. However, the analysis of large-scale samples across all age groups still provides valuable information about the dynamic EBV seropositivity with increasing age in Taiwan. Fourthly, this study was based on one public health survey conducted in 2007. Both serum samples and demographic data were collected at that time. Several relevant factors associated with the EBV seroprevalence, such as the ethnicity, household educational level, household income, and household size, were missed in the questionnaire and therefore were not analyzed in the study.

In summary, we clearly demonstrated that the seropositive rate of EBV significantly rose with the age in the Taiwanese population. The age-specific seropositive rate of EBV quickly increased from 23% at 0 year old to 53% at 2 years old. With the threat of acute EBV-associated infectious mononucleosis in childhood and the causal link between the EBV and certain malignancies in adults, vigorous prevention strategies, such as hygiene education, and the development of an effective vaccine, are needed to reduce the high infection rate in the susceptible population in Taiwan.

## Supporting Information

S1 Table The detailed data of demographics, potential risk factors for Epstein-Barr virus infection and serologic results of the 1411 study subjects. (XLSX)
